# TLSEA: a tool for lncRNA set enrichment analysis based on multi-source heterogeneous information fusion 

**DOI:** 10.3389/fgene.2023.1181391

**Published:** 2023-05-02

**Authors:** Jianwei Li, Zhiguang Li, Yinfei Wang, Hongxin Lin, Baoqin Wu

**Affiliations:** ^1^ Institute of Computational Medicine, School of Artificial Intelligence, Hebei University of Technology, Tianjin, China; ^2^ School of Electronic and Information Engineering, Hebei University of Technology, Tianjin, China

**Keywords:** lncRNA, functional enrichment analysis, heterogeneous network representation learning, lncRNA–lncRNA association network, random walk with restart, web server

## Abstract

Long non-coding RNAs (lncRNAs) play an important regulatory role in gene transcription and post-transcriptional modification, and lncRNA regulatory dysfunction leads to a variety of complex human diseases. Hence, it might be beneficial to detect the underlying biological pathways and functional categories of genes that encode lncRNA. This can be carried out by using gene set enrichment analysis, which is a pervasive bioinformatic technique that has been widely used. However, accurately performing gene set enrichment analysis of lncRNAs remains a challenge. Most conventional enrichment analysis methods have not exhaustively included the rich association information among genes, which usually affects the regulatory functions of genes. Here, we developed a novel tool for lncRNA set enrichment analysis (TLSEA) to improve the accuracy of the gene functional enrichment analysis, which extracted the low-dimensional vectors of lncRNAs in two functional annotation networks with the graph representation learning method. A novel lncRNA–lncRNA association network was constructed by merging lncRNA-related heterogeneous information obtained from multiple sources with the different lncRNA-related similarity networks. In addition, the random walk with restart method was adopted to effectively expand the lncRNAs submitted by users according to the lncRNA–lncRNA association network of TLSEA. In addition, a case study of breast cancer was performed, which demonstrated that TLSEA could detect breast cancer more accurately than conventional tools. The TLSEA can be accessed freely at http://www.lirmed.com:5003/tlsea.

## 1 Introduction

The central principle of molecular biology has proposed that RNA is an intermediary between protein-coding genes and proteins. However, genes encoding proteins only account for 1.5% of the human genome, and more than 98% of the human genome does not encode proteins. Most of these non-protein-coding genes were transcribed into non-coding RNAs (ncRNAs) (Guttman et al., 2009; Xue et al., 2017; DiStefano, 2018). These ncRNAs were often considered as “noise” of genome transcription and were not associated with any biological functions for decades. According to the length of the nucleotide sequence, ncRNAs can be further divided into small ncRNAs (<200 nucleotides) and long non-coding RNAs (>200 nucleotides, lncRNAs) (Chen et al., 2016; McDonel and Guttman, 2019). Although lncRNAs are not directly translated into proteins, their complex and diverse functions have helped gain insights into several biological processes in humans. As a novel class of ncRNAs, their functional studies received great interest, and considerable progress has been made in exploring lncRNA biology. Since the discovery of the lncRNAs H19 and XIST in the early 1990s (Tsang and Kwok, 2007; Li et al., 2013), substantial evidence has suggested that lncRNAs play an important role in the regulation of several processes, including epigenetic process, cell cycle, cell differentiation, and transcription mode (Gloss and Dinger, 2016; Kashi et al., 2016; Kopp and Mendell, 2018). With the rapid development of scientific methodology and experimental technology, researchers have identified thousands of lncRNAs that play important roles in many basic and key biological processes in eukaryotes, from nematodes to humans (Munos, 2009; Lalevée and Feil, 2015). Moreover, lncRNAs also play a key regulatory role in the occurrence and development of complex human diseases (Engreitz et al., 2016; Fang and Fullwood, 2016), such as breast cancer (Niknafs et al., 2016), non-small-cell lung cancer (Hua et al., 2019), gastric cancer (Liu et al., 2015), and cardiovascular diseases (Uchida and Dimmeler, 2015). Mutations or disorders of lncRNAs are closely related to many human diseases. For example, MALAT1 (or NEAT2) is upregulated in non-small-cell lung cancer and can be used as a biomarker for early cancer prognosis (Gutschner et al., 2013), and the use of lncRNA HOTAIR has been explored as a potential biomarker for detecting recurrence of hepatocellular carcinoma (Topel et al., 2020). With the advancement of high-throughput sequencing technology, more lncRNA gene sets have been produced as a result of data analysis using high-throughput experiments. However, it is still challenging to find how the associations between lncRNAs in one set can be used to develop a comprehensive understanding of the biological regulatory functions of lncRNA gene sets. Moreover, it is important to assess how the regulatory function of lncRNA lists of interest submitted by users on a large scale can be more accurately analyzed in the face of a large amount of omics data (Wang and Krishnan, 2014; Fillinger et al., 2019). We believe that the two aforementioned issues can be solved using the lncRNA set function enrichment analysis method, which identifies the importance of biological functions that are overrepresented in a long list with respect to their role in the whole human genome. The lncRNA set function enrichment analysis method has become an important research area in the field of lncRNA regulatory function research.

Gene set enrichment analysis was used to determine whether a group of genes with common characteristics (such as differential expression) were enriched on a certain functional pathway based on a gene set rather than a single gene, which would increase the reliability of gene function prediction (Del Giacco and Cattaneo, 2012). During the calculation, the gene set functional enrichment analysis method integrated data from different levels and sources and provided important insights for constructing characteristic gene modules and molecular regulatory networks in different physiological and pathological states. To date, dozens of gene set functional enrichment analysis methods have been developed that can be divided into four categories based on their data sources and execution algorithms.

The first category is over-representation analysis (ORA) methods, which are early and conventional enrichment analysis methods. Such methods intersect a group of genes of the user’s interest (called a gene list) with the background gene sets, count common genes as hit numbers, and evaluate whether the background gene set is significantly enriched in the gene list using statistical methods (Khatri et al., 2012). At present, there are many online tools and software that provide overexpression analysis, such as DAVID (Jiao et al., 2012), GOstats (Beissbarth and Speed, 2004), and GenMAPP (Doniger et al., 2003). In addition, LncSEA is an online tool that can enrich and analyze lncRNA lists using over-representation analysis methods. ORA methods are robust, reliable, and widely used. However, their limitations are also obvious, which hamper their application. The second category is functional class scoring (FCS) methods. Many FCS methods have been proposed, of which GSEA (Mootha et al., 2003; Subramanian et al., 2005) is the most commonly used one. FCS methods treat each lncRNA equally and in isolation, and the feature information of each gene within the background gene set and the associations with other genes are both neglected, which could be an obstacle to seeking more insightful biological processes for researchers. The third category is path topology (PT) methods. In the biological pathways, genes usually affect the biological processes of cells through complex relationships. Pathway-Express (Draghici et al., 2007) was the first PT method. SPIA (Tarca et al., 2009) introduced the concept of regulation intensity of each regulation relationship in a pathway based on retaining influencing factors. TopoGSA (Glaab et al., 2010) adopted the centripetality parameters of pathways while comparing differences between pathways. Currently, only the KEGG database (Kanehisa and Goto, 2000; Kanehisa et al., 2014) provides a comprehensive path topology. The fourth category is network topology (NT) methods. The key idea of NT methods is to convert the functional enrichment analysis problem of the gene list of interest into the functional enrichment analysis problem of gene pairs based on functional annotation networks. The most comprehensive and typical example of this category is the network ontology analysis (NOA) method (Wang et al., 2011). NT methods adopt gene importance and association information at the system level, overcome the defect that core genes are ignored due to small differential expression, and can make more accurate and reliable predictions. Therefore, NT methods are recommended when there are suitable gene function annotation networks, and they have become one of the mainstream methods associated with functional enrichment analysis at present.

Owing to the construction and integration of lncRNA-associated networks, NT methods based on gene network topology cannot be directly applied to lncRNA functional enrichment analysis. Inspired by the miRNA similarity network based on disease association, we constructed two lncRNA similarity networks based on miRNA–lncRNA associations and lncRNA–disease associations. These multi-source functional annotation networks provide an important basis for lncRNA functional enrichment analysis.

**FIGURE 1:**
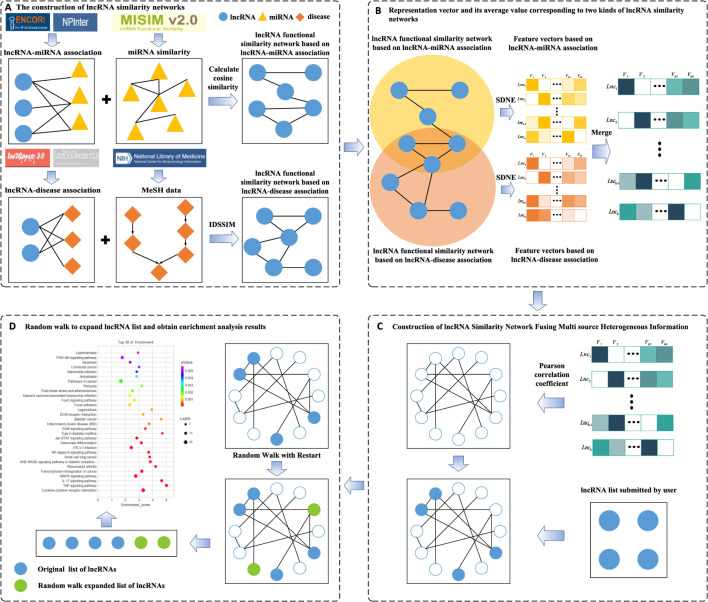
Flowchart of the TLSEA model. **(A)** Two lncRNA similarity networks were constructed based on lncRNA–miRNA associations and lncRNA–disease associations, respectively. **(B)** The SNDE method was used to extract 64-dimensional vectors from two networks, respectively. If an lncRNA appeared in two similar networks, its average value was taken as the final low-dimensional vector value. **(C)** The Pearson correlation coefficient was adopted to obtain the lncRNA similarity network that integrated multi-source heterogeneous information, and the differential expression list was used as the seed node of the network. **(D)** The expanded lncRNA list was obtained with network random walk with the restart method and was merged with the original lncRNA list; functional enrichment analysis was performed on it.

In this study, we aimed to develop a novel tool for lncRNA set enrichment analysis (TLSEA) to improve the accuracy of lncRNA set enrichment analysis. A flowchart of the TLSEA model is shown in Figure 1. First, two lncRNA functional similarity networks were constructed; the first network was based on lncRNA–miRNA associations by integrating the miRNA functional similarity network, and the second network was based on the lncRNA–miRNA association network and based on lncRNA–disease associations by integrating the disease semantic similarity network and lncRNA–disease association network, respectively. Second, to fuse a variety of heterogeneous lncRNA functional annotation networks and extract more feature information of lncRNA nodes, two sets of 64-dimensional vectors were created with the graph embedding algorithm structural deep network embedding (SDNE) based on the two lncRNA functional similarity networks and were merged into a new set of 64-dimensional vectors. If an lncRNA appeared in only one of the two functional annotation networks, its vector was retained as the merged vector. If one lncRNA appeared in both functional annotation networks, the average value of the two corresponding vectors was taken as the merged vector value. After merging, a feature matrix was obtained, in which each lncRNA node corresponded to a row vector. Third, the lncRNA–lncRNA association network was constructed by calculating the similarity of each corresponding lncRNA vector pair. Fourth, based on the lncRNA–lncRNA association network, a novel lncRNA functional enrichment analysis model could perform a more comprehensive and accurate enrichment analysis on the lncRNA list submitted by users from the two aspects of regulation function and disease association. It employed a network random walk with the restart method to enrich the lncRNA list prior to functional enrichment analysis. Our model mapped the nodes in the lncRNA list to the lncRNA–lncRNA association network as random walk seed nodes, and the lncRNA nodes closely associated with the subnet in the lncRNA–lncRNA association network were identified. Finally, both the lncRNA list submitted by the user and the expanded list were merged into a new lncRNA list, and functional enrichment analysis was performed.

## 2 Materials and methods

### 2.1 Datasets

Our study mainly included lncRNA–miRNA association data, lncRNA–disease association data, and lncRNA expression profile data. The lncRNA–miRNA association data, which were previously confirmed experimentally, were downloaded from the NPInter v4.0 (Teng et al., 2020) (see Supplementary Table S1) and ENCORI (Li et al., 2014) (see Supplementary Table S2) databases, and lncRNA symbols were obtained from the HGNC database (Seal et al., 2022). Thereafter, the lncRNA–miRNA association data obtained from the two databases were merged, and duplicate pairs were removed. LncRNA–miRNA pairs with non-standard naming formats were deleted, and the naming formats of both miRNA precursors and mature bodies were standardized. Finally, 18,033 validated lncRNA–miRNA associations were obtained between 1,002 lncRNAs and 437 miRNAs. The miRNA similarity data that were previously confirmed by experiments were downloaded from MISIM v2.0 (Li et al., 2019) and included 1,044 miRNAs (see Supplementary Table S3). The lncRNA–disease association data were downloaded from the LncRNADisease v2.0 (Bao et al., 2019) (see Supplementary Table S4) and Lnc2Cancer v3.0 (Gao et al., 2019) (Supplementary Table S5) databases. Thereafter, lncRNA functional similarity was calculated using disease semantic similarity; for this, all disease names were standardized according to the MeSH vocabulary (Baumann, 2016). The lncRNA–disease associations that did not conform to HGNC were removed. Finally, 2,230 validated lncRNA–disease associations were obtained, involving 777 lncRNAs and 257 diseases. LncRNA expression profile data were downloaded from the NONCODE database (Fang et al., 2018). After converting NONCODE ID to HGNC and removing lncRNAs with no expression, we obtained 303 lncRNAs from 24 tissues or organs.

### 2.2 Disease semantic similarity network

Using a previous method that was based on improved disease semantic similarity (Fan et al., 2020), we adopted IDSSIM, a model to calculate the functional similarity of lncRNAs in TLSEA. Primarily, IDSSIM introduced the IC contribution factor into the semantic value calculation, which considered both the hierarchical structure of the directed acyclic graph (DAG) and the specificities of diseases. IDSSIM was superior to conventional models, such as LNCSIM1, LNCSIM2 and ILNCSIM. No consideration of the hierarchical structure of the directed acyclic graph was included in LNCSIM1. ILNCSIM and LNCSIM2 considered only the specificities of diseases.

The semantic similarity between two diseases could be calculated using their DAG, which was constructed by mapping the names of the two diseases to MeSH names. For a disease A , its DAG is expressed as ${DAG}_{A}$ = { $T_{A}{,E}_{A}$ }, where $T_{A}$ is a collection of ancestor nodes of disease A and $E_{A}$ is the set of all edges in the DAG. The disease term $t\in T_{A}$ in ${DAG}_{A}$ had a semantic contribution to disease A , which was defined as the semantic value ${SV}_{A}^{1}\left(t\right)$ of t to disease A , calculated in LNCSIM1 (Chen et al., 2015) using the following formula:

$${SV}_{A}^{1}\left(t\right)=\left\{\begin{array}{c} 1\\ \max\left(∆\times {SV}_{A}^{1}\left(t^{\prime }\right)|t^{\prime }\in C\left(t\right)\right) \end{array}\right.\;\begin{array}{l} t=A\\ t\neq A \end{array},$$ (1)

where $C\left(t\right)$ is a subset of t and ∆ represents the semantic contribution factor of the edge connecting the linking disease term t with its child disease term $t^{\prime }$ in $E_{A}$ , which is usually set to 0.5 (Wang et al., 2010). In natural language processing, inverse document frequency is used to evaluate the importance of words in a document. From formula (1), we conclude that the higher the frequency of a disease, the lower its speech contribution.

In addition, LNCSIM2 adopts another common formula to calculate the contribution of the disease term $t\in T_{A}$ in ${DAG}_{A}$ to the semantic value ${SV}_{A}^{2}\left(t\right)$ of disease A :

$${SV}_{A}^{2}\left(t\right)=-\log\frac{DAGs\left(t\right)}{D},$$ (2)

where D is the number of diseases in MeSH and DAGs (t) is the number of DAGs that contain disease term t .

Using Equations 1, 2, the advantages of LNCSIM1 and LNCSIM2 methods were combined to calculate the semantic similarity of diseases. The contribution of the disease term t∈ $T_{A}$ in ${DAG}_{A}$ to the semantic value of disease A was calculated using the following equation:

$${SV}_{A}^{3}\left(t\right)=\left\{\begin{array}{c} 1\\ \max\left(\left(∆+P_{t}\right)\times {SV}_{A}^{3}\left(t^{\prime }\right)|t^{\prime }\in C\left(t\right)\right) \end{array}\right.\;\begin{array}{l} t=A\\ t\neq A \end{array}.$$ (3)

Here, $P_{t}$ is the IC contribution factor, which was calculated as follows:

$$P_{t}=\frac{\max_{k\in K}\left(DAGs\left(k\right)\right)-DAGs\left(t\right)}{D},$$ (4)

where K is the set of all the diseases in the MeSH. It should be noted that for disease term t , the $P_{t}$ value changed with the continuously updated versions of the MeSH.

The semantic value of disease A , $SV\left(A\right)$ , was then calculated as the sum of the contributions of all disease terms in ${DAG}_{A}$ to disease A :

$$SV\left(A\right)=\underset{t\in T_{A}}{\sum }{SV}_{A}^{3}\left(t\right).$$ (5)

Based on the intersection of the disease term set of diseases A and B , the semantic similarity of diseases A and B , $DSS\left(A,B\right)$ , was defined as follows:

$$DSS\left(A,B\right)=\frac{\underset{t\in T_{A}\cap T_{B}}{\sum }\left({SV}_{A}^{3}\left(t\right)+{SV}_{B}^{3}\left(t\right)\right)}{SV\left(A\right)+SV\left(B\right)},$$ (6)

where $T_{A}$ is a collection of ancestor nodes of disease A . $SV\left(A\right)$ is the sum of the contributions of all disease terms for disease A in ${DAG}_{A}$ . $DSS\left(A,B\right)$ is the disease semantic similarity between diseases A and B.

### 2.3 LncRNA functional similarity network based on lncRNA–miRNA associations

Previous studies have confirmed that lncRNAs with more common target miRNAs may have a higher similarity. Based on this assumption, an lncRNA functional similarity network was constructed by integrating lncRNA–miRNA association data with miRNA similarity data in the present study. LncRNA–miRNA association data were downloaded from the NPInter v4.0 and ENCORI databases, and miRNA similarity data were obtained from MISIM v2.0. We constructed lncRNA feature vectors based on lncRNA–miRNA associations and calculated the association scores of the two lncRNAs using cosine correlation.

**FIGURE 2:**
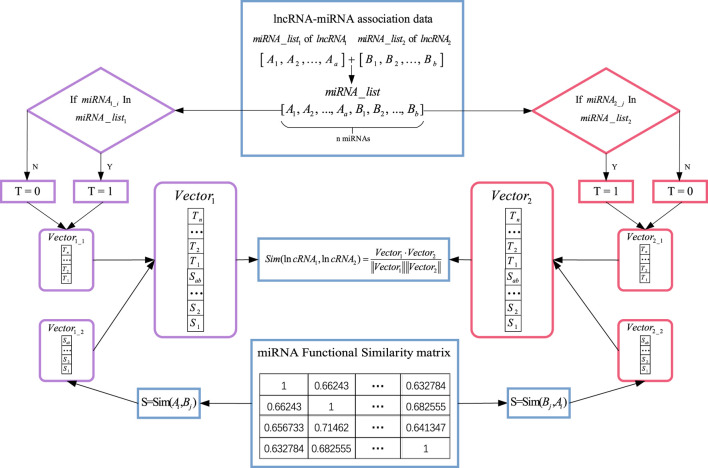
Flowchart of lncRNA similarity calculation based on lncRNA–miRNA associations.

As shown in Figure 2, we calculated the functional similarity between ${\ln cRNA}_{1}$ and ${lncRNA}_{2}$ by utilizing the data collected from the databases to generate the characteristics of the two lncRNAs based on the shared target miRNAs and the functional similarity between the target miRNAs. As associations between lncRNAs and miRNAs include multiple relationships, one lncRNA can target more than one miRNA, and conversely, multiple lncRNAs may target the same miRNA. Moreover, lncRNAs with the same target genes have generally similar functions (Tay et al., 2014). Based on this evidence, the miRNAs associated with ${lncRNA}_{1}$ and ${lncRNA}_{2}$ were first sorted into lists ${miRNA\_list}_{1}$ and ${miRNA\_list}_{2}$ , respectively. After removing duplicate elements, ${miRNA\_list}_{1}$ and ${miRNA\_list}_{2}$ were merged to create a new list $miRNA_{list}$ , which contained all miRNAs interacting with *lncRNA*_*1*_ and *lncRNA*_*2*_. The number of miRNA_list was n. Then, two vectors, ${Vector}_{1\_1}$ and ${Vector}_{2\_1}$ , were utilized as the first parts of the feature vectors of ${lncRNA}_{1}$ and ${lncRNA}_{2}$ to describe the common target miRNAs of ${lncRNA}_{1}$ and ${lncRNA}_{2}$ . For each miRNA in the miRNA_list , if it existed in the ${miRNA\_list}_{1}$ , T was 1, and otherwise, 0. The values of T were added to ${Vector}_{1\_1}$ and ${Vector}_{2\_1}$ . Thus, ${Vector}_{1\_1}$ and ${Vector}_{2\_1}$ are vectors composed of 1 and 0. The more the two lncRNAs that had common target miRNAs, the more the elements with the same position in ${Vector}_{1\_1}$ and ${Vector}_{2\_1}$ had the same value of 1. The higher the similarity between two vectors, the higher their functional similarity. Subsequently, miRNAs in ${miRNA\_list}_{1}$ and ${miRNA\_list}_{2}$ were extracted to form miRNA set A and miRNA set B , and the similarity values of $Sam\left(a_{i},B\right)$ and $Sam\left(b_{j},A\right)$ were calculated to form ${Vector}_{1\_2}$ and ${Vector}_{2\_2}$ , respectively. ${Vector}_{1\_2}$ and ${Vector}_{2\_2}$ denote the functional similarity of the miRNAs that were targeted by ${lncRNA}_{1}$ and ${lncRNA}_{2}$ . The higher the functional similarity of the associated miRNAs, the higher the similarity between ${Vector}_{1\_2}$ and ${Vector}_{2\_2}$ . Finally, ${Vector}_{1\_1}$ and ${Vector}_{1\_2}$ are merged into ${Vector}_{1}$ . Following this approach, ${Vector}_{2}$ was created. Ultimately, ${Vector}_{1}$ and ${Vector}_{2}$ represented ${lncRNA}_{1}$ and ${lncRNA}_{2}$ , respectively. The cosine similarity formula was adopted to calculate the functional similarity scores of the ${lncRNA}_{1}$ and ${lncRNA}_{2}$ as follows:

$$Sim\left({lncRNA}_{1},{lncRNA}_{2}\right)=\frac{{Vector}_{1}∙{Vector}_{2}}{\left\|{Vector}_{1}\right\|\left\|{Vector}_{2}\right\|}.$$ (7)

### 2.4 LncRNA functional similarity network based on lncRNA–disease associations

**FIGURE 3:**
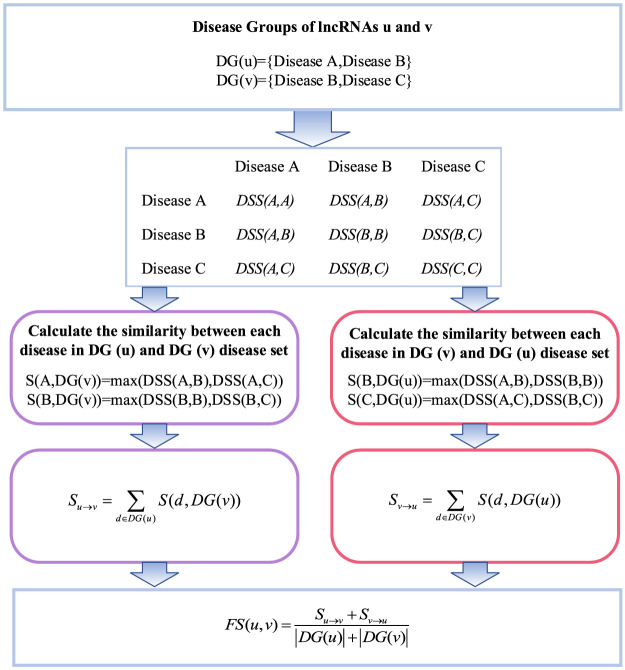
Flowchart of lncRNA function similarity calculation.

The calculation of lncRNA functional similarity is that lncRNAs related to similar diseases may have similar functions. LncRNA functional similarity can be calculated by integrating the semantic similarity of diseases and known lncRNA–disease association data. The flowchart of lncRNA functional similarity based on lncRNA–disease associations is shown in Figure 3, where $DG\left(u\right)$ and $DG\left(v\right)$ were defined as all disease sets related to lncRNA u and lncRNA v , respectively. The semantic similarity of each disease in $DG\left(u\right)$ and $DG\left(v\right)$ was used to calculate the lncRNA functional similarity between lncRNA u and lncRNA v .

Specifically, the similarity coefficient between one disease in the disease set corresponding to lncRNA u and all disease sets of lncRNA v was first calculated as follows:

$$S\left(d_{u},DG\left(v\right)\right)=\max_{d\in DG\left(v\right)}\left(DSS\left(d_{u},d\right)\right),$$ (8)

$$S\left(d_{v},DG\left(u\right)\right)=\max_{d\in DG\left(u\right)}\left(DSS\left(d_{v},d\right)\right),$$ (9)

where $d_{u}$ and $d_{v}$ represent disease in $DG\left(u\right)$ and $DG\left(v\right)$ , DSS is the disease semantic similarity, and $S\left(d_{u},DG\left(v\right)\right)$ is the similarity between disease $d_{u}$ and disease group $DG\left(v\right)$ .

Thereafter, the coefficients of the disease set of lncRNA u and the disease set of lncRNA v were accumulated as

$$S_{u\to v}=\underset{d\epsilon DG\left(u\right)}{\sum }S\left(d,DG\left(v\right)\right),$$ (10)

$$S_{v\to u}=\underset{d\epsilon DG\left(v\right)}{\sum }S\left(d,DG\left(u\right)\right).$$ (11)

Finally, the functional similarity between lncRNA u and lncRNA v was defined as

$$FS\left(u,v\right)=\frac{S_{u\to v}+S_{v\to u}}{\left|DG\left(u\right)\right|+\left|DG\left(v\right)\right|},$$ (12)

where the operator $\left|.\right|$ represents the total number of diseases corresponding to the disease sets.

### 2.5 LncRNA functional similarity network based on expression profiles

Functionally interacting genes tend to exhibit similar expression profiles, thereby providing a theoretical basis for the calculation of lncRNA similarity with lncRNA expression profile data. Therefore, lncRNA functional similarity imputation methods based on expression profiles have been used in lncRNA function research. Analysis of lncRNA characteristics indicated that lncRNAs have significant tissue specificity and are conserved in mammals. The expression profiles of lncRNAs vary between different tissues and change at different growth stages in the same tissue or organ (Zhu et al., 2014). In the present study, the lncRNA expression profiles of 24 tissues and organs were downloaded from the NONCODE database. Each item had 24 dimensions, representing the expression profiles of this lncRNA in 24 tissues or organs. In the present study, some items missing the expression profile were deleted, and the naming format of the lncRNA was standardized. Finally, 303 lncRNA expression profiles were obtained. The correlation between these lncRNAs was analyzed using the Spearman correlation analysis method, and the Spearman correlation coefficient between two lncRNAs was adopted to determine their similarity.

### 2.6 Graph embedding methods

Currently, many graph embedding methods have been proposed to discover novel proper mapping functions to convert graph data that are usually high-dimensional sparse matrices to low-dimensional dense vectors. They maintained the proximity of these low-dimensional vector representations to solve the conundrum, which was difficult to consider using machine learning algorithms. Hence, graph embedding methods, such as node classification, link prediction, and association mining, have been used for mining biological information.

Existing graph embedding models are generally divided into five categories according to their algorithm principles: graph embedding based on matrix decomposition, graph embedding based on random walk, graph embedding based on self-encoder, graph embedding based on graph neural networks (GNNs) (Scarselli et al., 2009), and graph embedding based on other methods. In our study, we adopted four types of prevailing graph embedding algorithms to obtain low-dimensional dense vectors of graph data: DeepWalk and Struc2Vec (based on random walk), SDNE (based on self-encoder), and LINE (based on other methods).

DeepWalk is a graph embedding method based on Word2vec. It is an extension of the language model and unsupervised learning from word sequences to graphs. First, the neighbor nodes of the nodes in the network were randomly generated to form a fixed-length random walk sequence, and then, the generated fixed-length node sequence was mapped into a low-dimensional embedded vector using the skip-gram model. The generated vector encoded the relationship between nodes in the low-dimensional vector space, which was used to capture neighborhood similarity and community structure and extracted the potential characteristics of the nodes. This method can learn the relationship information of node pairs and realize incremental learning of dynamic graphs; its time complexity is $O\left(\log\left|V\right|\right)$ . However, the performance of this method in a weighted graph was poor, as it could only maintain the second-order similarity of the graph, and the explicit objective function was not used in the optimization process, which limited the ability of the model to maintain the network structure, which would affect the integrity of the context information.

SDNE utilized the depth self-encoder and the first-order and second-order similarities of the graph to obtain the final embedded vectors through the highly non-linear function and the optimization objective function, which can effectively capture the highly non-linear network structure. SDNE includes supervised and unsupervised components that maintain the first-order and second-order similarities of the nodes. The supervised component introduced Laplacian feature mapping as the objective function of first-order similarity so that the generated embedding can capture local structure information. The unsupervised component modifies the L2 reconstruction loss function as the objective function of the second-order similarity so that the generated embedding can obtain the global structural features. The joint optimization of the first- and second-order similarities enhanced the robustness of the model on the sparse graph, and the generated embedding preserved the global and local structure information simultaneously. However, SDNE was inefficient in realizing the embedding of network nodes with higher orders of magnitude and could not realize the incremental update of graphs.

LINE also defined and optimized first-order and second-order similarity functions. First-order similarity was used to keep the point product of the adjacency matrix close to the embedded representation, and second-order similarity was adopted to maintain the similarity of the context nodes. LINE optimized the objective functions of the first-order and second-order similarities to minimize the distance between the node pair probability distribution generated by the adjacency matrix and the probability distribution generated by the embedded inner product through KL divergence, realized the optimization of graph embedding, and spliced the generated embedding vectors. The edge sampling strategy of LINE overcomes the limitations of random gradient descent and makes it applicable to large-scale graph embedding. However, the single optimization of the first-order and second-order representations and the simple splicing operation also limit the representation ability of LINE.

Unlike conventional graph embedding models, Struc2Vec focuses on the roles of different nodes in the network. The features of the nodes represent their locations and relationships with other nodes. However, many existing algorithms only express the nodes as vectors according to the distance relationships and do not consider the other structural features of the nodes. Most graph embedding models believe that the more common the neighbors of two nodes, the more similar the two nodes are, and it is natural to reduce their distance in the embedding space. However, this method cannot be used to identify node pairs with similar structures. In fact, some nodes have similar topological structures, but are too far away to have common neighbors. Struc2Vec ignores the attributes of nodes and edges and their positions in the network to evaluate the structural similarity between nodes; however, the limitations were still present.

### 2.7 Server construction

In this study, we developed a web server named TLSEA for lncRNA functional enrichment analysis, which was based on the fusion of heterogeneous information obtained from multiple sources. The flask framework was adopted for data processing and calculation. At the front end of the TLSEA, the framework of “HTML + CSS + Bootstrap” was employed, and Ploty.js and JQuery were used for graphical visualization and application logic, respectively. All computational algorithms were implemented in Python using the NumPy and Pandas packages. In total, lncRNA pathways of 385 diseases were identified (see Supplementary Table S6). TLSEA is unrestricted (without a login procedure), compatible with most web browsers, and accessible at http://www.lirmed.com:5003/tlsea.

## 3 Results

### 3.1 LncRNA feature vector selection

**FIGURE 4:**
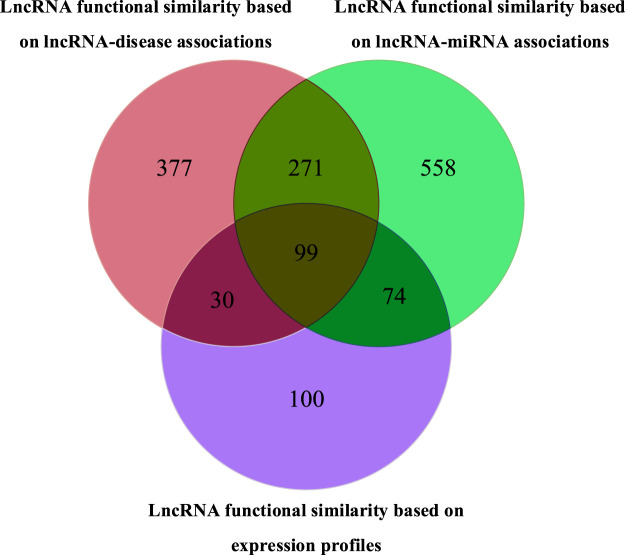
Venn diagram of three lncRNA sets in lncRNA feature vector selection.

Three lncRNA networks were constructed using different similarity networks, as shown in Figure 4. The first lncRNA functional similarity network was constructed by integrating lncRNA–miRNA association data and miRNA similarity data, which included 1,002 lncRNAs (named LncRNAset1). The second lncRNA functional similarity network was constructed by integrating lncRNA–disease association data and disease semantic similarity network and included 777 lncRNAs (named LncRNAset2) and the third lncRNA functional similarity network by lncRNA expression profile data, which included 303 lncRNAs (named LncRNAset3).

To gain better low-dimensional feature vectors of lncRNAs, we extracted 16-, 32-, 64-, and 128-dimensional feature vectors with four types of prevailing graph embedding algorithms (DeepWalk, Struc2Vec, SDNE, and LINE). In this study, a random forest classifier was selected to evaluate the performance of the four algorithms. LncRNAset1, LncRNAset2, and LncRNAset3 were fed into the classifier to train it. Four types of random forest classifiers, namely, R1(X), R2(X), R3(X), and R4(X), were employed based on the 16-, 32-, 64-, and 128-dimensional lncRNA feature vectors extracted by the DeepWalk, Struc2Vec, SDNE, and LINE methods in turn. Finally, the effectiveness of each type of lncRNA feature vector was validated using 10-fold cross validation. Accuracy (ACC) values are listed in Table 1. The numbers in the vector names represent feature vector dimensions. For example, DeepWalk16 represents the DeepWalk algorithm with 16-dimensional feature vectors.

**TABLE 1:** ACC results of different lncRNA feature vectors.

Types of lncRNA feature vectors	ACC based on lncRNA–disease association	ACC based on lncRNA–miRNA association
DeepWalk16	0.8638	0.8066
LINE16	0.8869	0.8184
SDNE16	0.9815	0.9078
Struc2Vec16	0.8588	0.8246
DeepWalk32	0.8467	0.8101
LINE32	0.9351	0.8220
SDNE32	0.9812	0.9061
Struc2Vec32	0.8693	0.8186
DeepWalk64	0.8854	0.8000
LINE64	0.9509	0.8063
SDNE64	0.9824	0.9131
Struc2Vec64	0.8840	0.7996
DeepWalk128	0.8907	0.7324
LINE128	0.9559	0.7888
SDNE128	0.9822	0.9045
Struc2Vec128	0.8931	0.7380

As shown in Table 1, the feature vectors extracted by SDNE64 yielded the best classification results for the two lncRNA functional similarity networks of lncRNA feature vectors, which are marked in bold. Therefore, we believe that the 64-dimensional vector extracted by SDNE could retain more information and be selected in this study.

### 3.2 Construction of an lncRNA–lncRNA association network

First, two sets of 64-dimensional lncRNA feature vectors based on the lncRNA–miRNA association network and lncRNA–disease association network were merged into a new set. If one lncRNA appeared in only one of the two association networks, its vector was retained as the merged vector. If it appeared in both functional annotation networks, the average value of the two corresponding vectors was considered as the merged vector value. After merging, a novel lncRNA feature matrix was obtained in which each lncRNA node of the two association networks corresponded to a row vector. The Pearson correlation coefficient ρ was used to evaluate the closeness of the relationship between two lncRNA feature vectors, which was calculated using the following formula:

$$\rho =\frac{\sum_{i=1}^{N}\left(x_{i}-\bar{x}\right)\left(y_{i}-\bar{y}\right)}{{\left[\sum_{i=1}^{N}{\left(x_{i}-\bar{x}\right)}^{2}\sum_{i=1}^{N}{\left(y_{i}-\bar{y}\right)}^{2}\right]}^{\frac{1}{2}}},$$ (13)

where $\bar{x}$ is the average values for all x and $\bar{y}$ is the average values for all y .

Integrating the lncRNA functional similarity based on lncRNA–disease association and lncRNA functional similarity based on lncRNA–miRNA association network, an lncRNA–lncRNA association network (see Supplementary Table S7) was constructed by merging lncRNA-related heterogeneous information obtained from multiple sources and various lncRNA-related similarity networks, which included 1,409 lncRNAs.

### 3.3 Overview of the TLSEA web server

**FIGURE 5:**
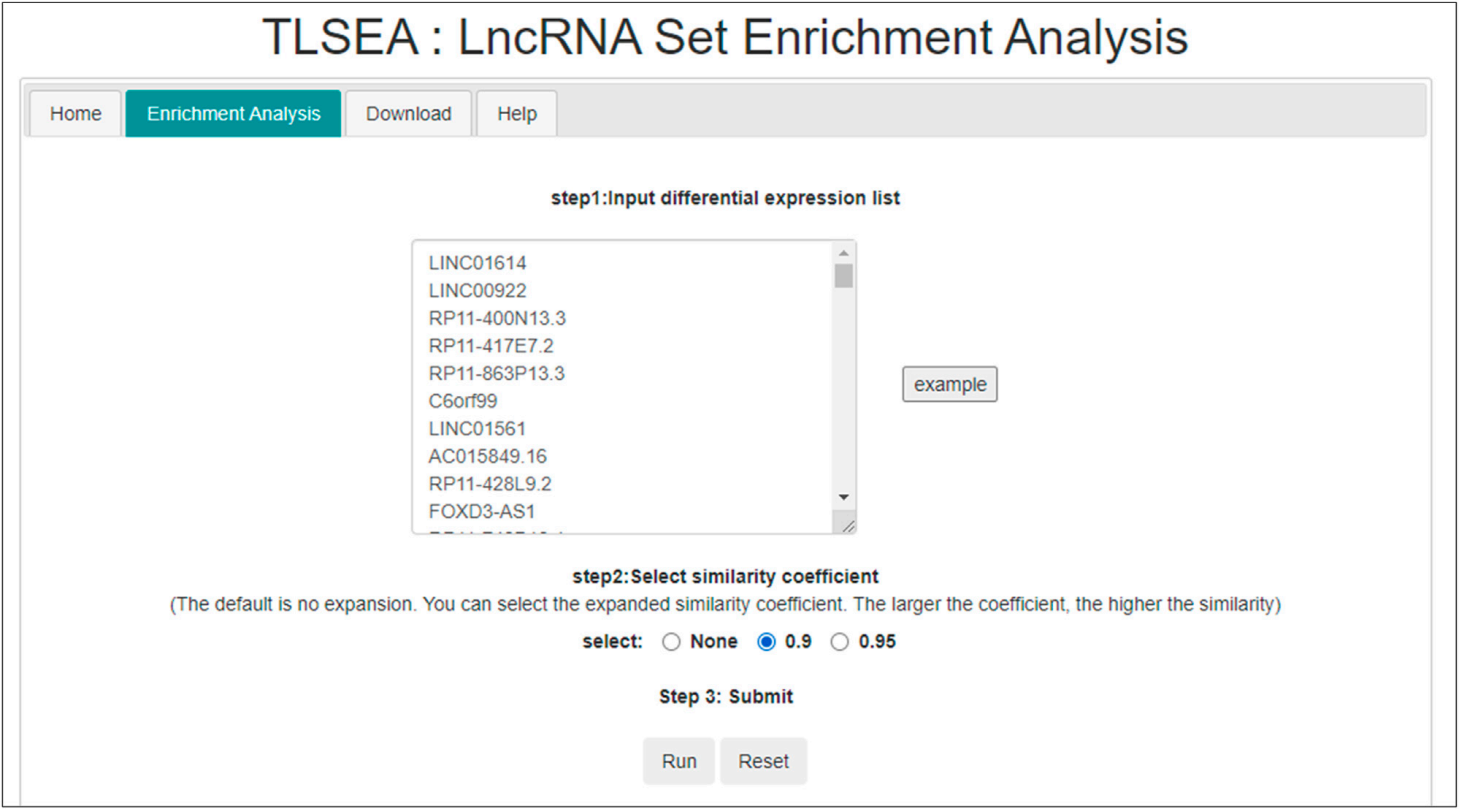
Enrichment analysis page of TLSEA.

**FIGURE 6:**
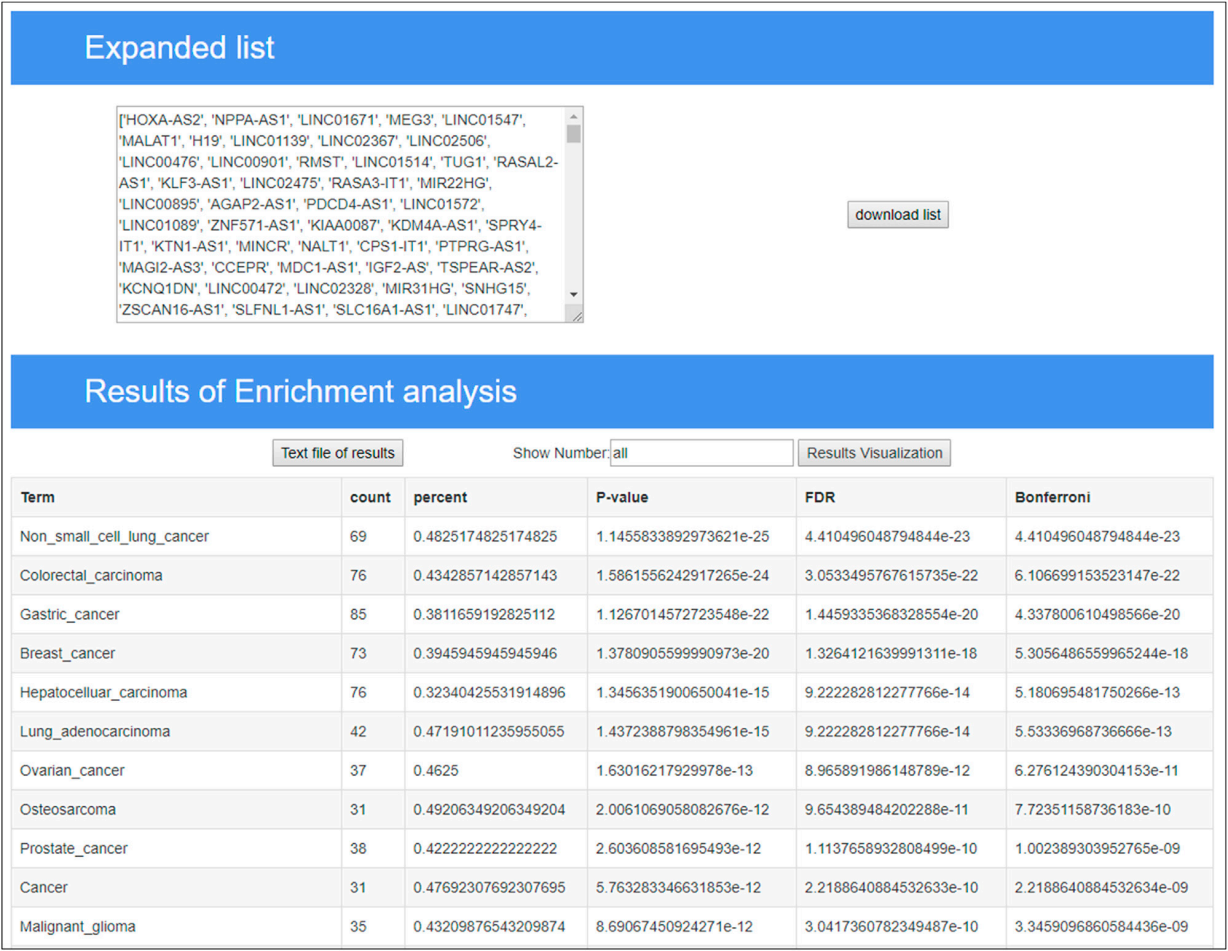
Enrichment analysis result page of TLSEA.

In the TLSEA, users only need to submit a list of lncRNAs of interest. Users can utilize the TLSEA to calculate the *p*-values of the original lncRNA list and the lncRNA list after random walk expansion. As shown in Figures 5, 6, the web interface of the TLSEA was designed according to the following workflow. First, the user inputs an lncRNA list of interest and then selects the similarity coefficient for expansion. The larger the similarity coefficient, the more similar the expanded lncRNA list is to the original lncRNA list. The unified lncRNA naming format used in the TLSEA enrichment analysis is the HGNC symbol. If the lncRNA names did not match the HGNC symbols, users needed to convert them to this format using the LncBook 2.0 database (Li et al., 2022) or other conversion tools before analyzing the data with the TLSEA. If the user chooses the similarity coefficient as “None,” it implies that only the original lncRNA list was used for enrichment analysis. Finally, the user clicked the “Run” button to complete the task. If the similarity coefficient was not “None,” the TLSEA would additionally display the expanded lncRNA list and provide a button to export it. The enrichment analysis results are shown in Figure 6. TLSEA could also visualize the results with one bubble chart by clicking the “Results Visualization” button.

### 3.4 Case studies

To further evaluate the application of the TLSEA model in practical situations, we used the TLSEA to analyze the functions of differentially expressed genes in breast cancer. For the case study, we first downloaded a list of differentially expressed lncRNAs ( $\log_{2}FC>1$ ; $P_{adj}<0.05$ ) of breast cancer from the TCGA project and CircRNAnet database as the input list of the TLSEA. Then, the similarity coefficient was set to “None,” which meant that the enrichment analysis was performed based on the original differential expression lncRNA list, and the “Run” button was clicked to implement the enrichment analysis. The TLSEA would output all disease pathways with a *p*-value <0.01 and provide the results of visual enrichment analysis. The top 15 significances of the enrichment analysis results are displayed on the results page. Subsequently, the similarity coefficient was set to 0.9, which meant that the TLSEA would screen the lncRNA–lncRNA association network based on multi-source heterogeneous information fusion in advance, retaining only the edges whose similarity values exceeded 0.9. Subsequently, the differentially expressed lncRNAs were used as seed nodes, and the random walk with restart method was performed on the lncRNA–lncRNA association network. After all nodes converged, the nodes whose random walk probabilities were not 0 were identified as expanded lncRNAs and used to obtain the expanded lncRNA list. Finally, the TLSEA performed an enrichment analysis of these expanded lncRNAs.

**FIGURE 7:**
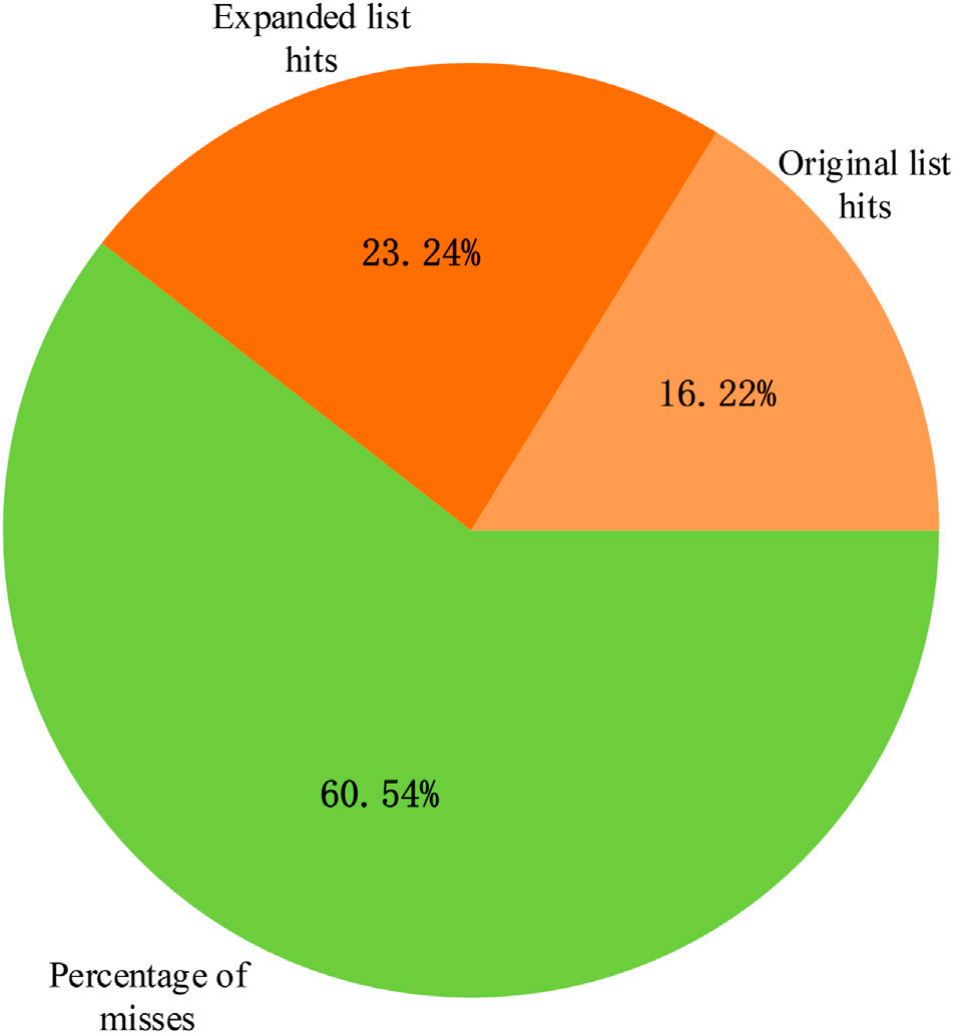
Percentage of lncRNA hit terms after performing random walk with the restart method.

The breast cancer disease lncRNA set included 185 lncRNAs and only 30 lncRNAs from the original differentially expressed lncRNA list, with a hit rate of 16.22%. The case study results showed that 73 lncRNAs from the expanded lncRNA list were hit after performing the random walk with the restart method, and the hit rate increased to 39.46%, as shown in Figure 7. The *p*-value of breast cancer with the original differentially expressed lncRNA list was $1.34e^{-13}$ , and the *p*-value of breast cancer with the expanded list after performing random walk with the restart method was $1.37e^{-20}$ . The expanded list was significantly enriched in breast cancer compared with the original list. In addition, the *p*-values of the top 10 diseases in the enrichment analysis results of the original list were significantly improved, as shown in Table 2. Experimental findings proved that TLSEA could effectively improve the accuracy of enrichment analysis of the lncRNA list.

**TABLE 2:** Comparison of *p*-values of the origin list and expanded list of the top 10 diseases in TLSEA.

Disease	*p*-values of the origin list	*p*-values of the expanded list
Non-small-cell lung cancer	$5.48e^{-14}$	$1.15e^{-25}$
Breast cancer	$1.34e^{-13}$	$1.38e^{-20}$
Gastric cancer	$6.27e^{-13}$	$1.13e^{-22}$
Colorectal carcinoma	$5.26e^{-11}$	$1.59e^{-24}$
Nasopharyngeal carcinoma	$2.00e^{-8}$	$4.85e^{-10}$
Prostate cancer	$2.49e^{-8}$	$2.60e^{-12}$
Esophageal squamous cancer	$3.64e^{-8}$	$2.33e^{-11}$
Thyroid cancer	$6.52e^{-8}$	$3.00e^{-8}$
Esophageal cancer	$1.49e^{-7}$	$3.15e^{-8}$
Squamous cell carcinoma	$1.87e^{-7}$	$1.19e^{-7}$

After statistical analysis, 43 additional lncRNAs were calculated based on the expanded list, which were not found based on the original lncRNA list. All of them were validated in the literature; their names and corresponding PMIDs are listed in Table 3.

**TABLE 3:** List of 43 additional lncRNAs and corresponding PMIDs of breast cancer based on the expanded lncRNA list.

lncRNA	PMID	lncRNA	PMID
HOXA-AS2	28545023	LINC02099	27597120
LINC00472	33668040	CERNA2	32248842
NORAD	34190442	SNHG15	32141559
SNHG7	33099915	MIR31HG	34076993
RMST	29215701	HIF1A-AS2	30635931
CYTOR	33842324	LINC00636	26929647
RASSF1-AS1	31062660	LINP1	27111890
FGF14-AS2	31486497	EGOT	26159853
NNT-AS1	32691576	PTPRG-AS1	34326372
LINC00598	28339037	LINC00901	25435812
NBAT1	26378045	CASC2	29523222
DIRC3	25122612	MALAT1	30349115
LINC01089	31417284	MAGI2-AS3	32730644
STXBP5-AS1	34764730	LINC-ROR	29041978
SNHG16	32122142	MEG3	33845141
TUG1	33380806	SPRY4-IT1	31736268
IRAIN	25465188	H19	33324070
FOXC2-AS1	29562954	JADRR	24097061
LINC02130	28003470	CASC22	24879036
LINC00339	31781497	PDCD4-AS1	33248413
KLF3-AS1	29453409	LINC01671	28003470
LINC00993	31921620		

The enrichment results calculated by the TLSEA not only significantly improved the *p*-values of the diseases in the enrichment results but also mined new diseases that were not enriched by the original lncRNA list. In this study, the expanded lncRNA list of breast cancer was significantly enriched in head and neck squamous cell carcinoma, with a *p*-value of $1.38e^{-6}$ . According to the lncSEA database (Chen et al., 2021), there were a total of 12 lncRNAs related to head and neck squamous cell carcinoma, and nine of them were hit in our case study. In contrast, only two lncRNAs were hit based on the original lncRNA list, as shown in Table 4. The experimental results of this case have previously shown that there is a certain relationship between breast cancer and head and neck squamous cell carcinoma (Croce, 2022), which also indicates that the TLSEA can detect some new diseases ignored by conventional enrichment analysis methods.

**TABLE 4:** Enrichment analysis results of the original lncRNA list and the expanded lncRNA list on head and neck squamous cell carcinoma.

lncRNA	PMID	Original list	Expanded list
C5orf66-AS1	30280186	Hitting	Hitting
CYTOR	35963855	Not hitting	Hitting
SPRY4-IT1	29575229	Not hitting	Hitting
FAM3D-AS1	Unconfirmed	Not hitting	Hitting
HAND2-AS1	29575229	Not hitting	Hitting
H19	27994496	Not hitting	Hitting
LUCAT1	29575229	Not hitting	Hitting
HEIH	29575229	Not hitting	Hitting
HOTAIR	31297902	Hitting	Hitting
EPB41L4A-AS2	29490660	Not hitting	Not hitting
LNC-JPH1-7	27323410	Not hitting	Not hitting
LNC-LCE5A-1	25904139	Not hitting	Not hitting

## 4 Discussion

Gene set enrichment analysis is a pervasive bioinformatic technique used to detect the underlying biological pathways and functional categories of a given gene list. In this study, we developed an lncRNA set enrichment analysis tool, the TLSEA, based on a multi-source heterogeneous information fusion. Conventional algorithms, such as hypergeometric and binomial tests, do not explicitly consider the rich association information among input lncRNAs in the lncRNA list, which is a hindrance in obtaining more insightful biological processes. We introduced the interaction information between miRNAs and lncRNAs and that between diseases and lncRNAs to construct a novel lncRNA–lncRNA association network and expanded the lncRNA list using random walk with restart. Using a case study, TLSEA demonstrated that the expanded lncRNA list can detect more insightful pathways than the original lncRNA list. Additionally, TLSEA provides a simple and user-friendly interface for analyzing, browsing, and downloading detailed information from lncRNA set enrichment analysis, which can help researchers understand the mechanisms of disease and develop effective diagnosis and treatment.

Generally, the expanded lncRNA list is more significantly enriched in the corresponding disease pathway than the original lncRNA. However, sometimes, selecting a small threshold for the similarity coefficient may lead to the introduction of some unrelated lncRNAs, which would cause poor enrichment analysis results. Therefore, how to provide a more suitable expansion strategy for users will be the future subject of a follow-up article on TLSEA. In addition, the insufficient disease pathway data will limit the comprehensiveness of enrichment analysis and calculation results. As the size of disease pathway data grows, TLSEA will supplement and include more multi-source heterogeneous information on lncRNAs. The future version of TLSEA will include more categories of lncRNAs and integrate additional functional information into the knowledge base, annotate more species, and develop a more efficient expanding method for lncRNA lists of interest.

## Data Availability

The original contributions presented in the study are included in the article/Supplementary Material; further inquiries can be directed to the corresponding author.
